# Physiological Ca^2+^ Transients Versus Pathological Steady-State Ca^2+^ Elevation, Who Flips the ROS Coin in Skeletal Muscle Mitochondria

**DOI:** 10.3389/fphys.2020.595800

**Published:** 2020-10-22

**Authors:** Ang Li, Jianxun Yi, Xuejun Li, Jingsong Zhou

**Affiliations:** Department of Kinesiology, College of Nursing and Health Innovation, The University of Texas at Arlington, Arlington, TX, United States

**Keywords:** skeletal muscle, mitochondrial ROS, mitoflash, mitochondrial Ca^2+^ homeostasis, transitory mPTP opening, electric field stimulation

## Abstract

Mitochondria are both the primary provider of ATP and the pivotal regulator of cell death, which are essential for physiological muscle activities. Ca^2+^ plays a multifaceted role in mitochondrial function. During muscle contraction, Ca^2+^ influx into mitochondria activates multiple enzymes related to tricarboxylic acid (TCA) cycle and oxidative phosphorylation, resulting in increased ATP synthesis to meet the energy demand. Pathophysiological conditions such as skeletal muscle denervation or unloading also lead to elevated Ca^2+^ levels inside mitochondria. However, the outcomes of this steady-state elevation of mitochondrial Ca^2+^ level include exacerbated reactive oxygen species (ROS) generation, sensitized opening of mitochondrial permeability transition pore (mPTP), induction of programmed cell death, and ultimately muscle atrophy. Previously, both acute and long-term endurance exercises have been reported to activate certain signaling pathways to counteract ROS production. Meanwhile, electrical stimulation is known to help prevent apoptosis and alleviate muscle atrophy in denervated animal models and patients with motor impairment. There are various mechanistic studies that focus on the excitation-transcription coupling framework to understand the beneficial role of exercise and electrical stimulation. Interestingly, a recent study has revealed an unexpected role of rapid mitochondrial Ca^2+^ transients in keeping mPTP at a closed state with reduced mitochondrial ROS production. This discovery motivated us to contribute this review article to inspire further discussion about the potential mechanisms underlying differential outcomes of physiological mitochondrial Ca^2+^ transients and pathological mitochondrial Ca^2+^ elevation in skeletal muscle ROS production.

## Introduction

Skeletal muscle carries out multiple critical functions of human body such as locomotion, metabolism, and thermogenesis (Block, 1994; Qiu et al., 2018). Thus, skeletal muscle atrophy, characterized by loss of muscle mass and strength, could have severe impact on daily living or even become life-threatening (Jackman and Kandarian, 2004). Human and animal studies revealed a variety of etiological factors for skeletal muscle atrophy, including disuse (limb immobilization, unloading) (Jackman and Kandarian, 2004), denervation (spinal motor neuron lesion in patients or surgical transection of motor nerves in animals) (Hoellwarth and Christian Hofer, 2005; Adhihetty et al., 2007), fasting (Qiu et al., 2018), lack of gravity (Fitts et al., 2001), aging (sarcopenia) (Dupont-Versteegden, 2005), cancer (cachexia) (Tisdale, 2010), neuromuscular diseases such as amyotrophic lateral sclerosis (ALS), and spinal muscular atrophy (Fischer et al., 2004; Monani, 2005). Accumulating evidence highlights programmed cell death (apoptosis) as a major cause of muscle fiber loss in skeletal muscle atrophy (Borisov and Carlson, 2000; Tews, 2002; Siu and Alway, 2005; Adhihetty et al., 2007), potentially implicating mitochondrial abnormality as a shared pathological feature underlying muscle atrophy induced by different etiological factors.

Mitochondria take up around 10–15% volume of mammalian skeletal muscle fibers (Eisenberg, 2010), with a certain degree of compositional and function differences observed between the subsarcolemmal, interfibrillar and peri-nuclear subpopulations (Cogswell et al., 1993; Díaz-Vegas et al., 2019). Mitochondria, especially the interfibrillar subpopulation, not only serve as the primary energy provider but also are intimately involved in apoptosis (Adhihetty et al., 2005; Siu and Alway, 2005; Chabi et al., 2008; Wang and Youle, 2009). The connections between mitochondria and apoptosis include:

1. Multiple proapoptotic proteins are located within mitochondria, such as cytochrome c (Cyto c) (Liu et al., 1996), apoptosis-inducing factor (AIF) (Susin et al., 1999), second mitochondria-derived activator of caspase/direct IAP (inhibitor-of apoptosis) binding protein (Smac/Diablo) (Du et al., 2000), Endonuclease G (EndoG) (Li et al., 2001), and high temperature requirement protein A2 (HtraA2) (Hegde et al., 2002).

2. Although healthy mitochondria are not the major contributor to cytosolic ROS in skeletal muscle during contractile activities (Sakellariou et al., 2013; Powers et al., 2016; Henríquez-Olguin et al., 2019), they can significantly contribute to ROS production under pathological conditions (Pottecher et al., 2013; Lejay et al., 2014). Upon overwhelming the cellular antioxidants’ neutralizing capacity, ROS causes oxidative damages to lipids, proteins, and DNA (Bandyopadhyay et al., 1999). Elevated ROS production is frequently observed as an early event of the apoptotic process (Fernandez et al., 2002).

3. Long-term elevation of mitochondrial matrix Ca^2+^ ([Ca^2+^]_mito_) can induce cell apoptosis through increasing the release of proapoptotic proteins and mitochondrial permeability transition pore (mPTP) opening (Haworth and Hunter, 1979; Hirsch et al., 1997; De Giorgi et al., 2002; Borutaite et al., 2003). It is worth noticing that interfibrillar mitochondria are more prone to release proapoptotic factors upon ROS stimulation, potentially due to higher probability of mPTP opening than subsarcolemmal mitochondria (Adhihetty et al., 2005). The detailed mechanisms will be discussed later in this review.

Although long-term [Ca^2+^]_mito_ elevation is closely associated with excessive ROS production (Adam-Vizi and Starkov, 2010; Peng and Jou, 2010), we observed an interesting phenomenon that mitochondrial Ca^2+^ transients induced by the electrical stimulation can decrease ROS production in denervated skeletal muscle fibers within a minute (Karam et al., 2017). This phenomenon is different from the excitation–transcription coupling events that help skeletal muscle cope with ROS during exercise or electrical stimulation, which usually occur in the time frame of hours or longer (Tsuboyama-Kasaoka et al., 1998; Cortright et al., 1999; Pilegaard et al., 2003; Egan et al., 2010). Although the underlying molecular mechanism of the instant ROS suppression by the rapid mitochondrial Ca^2+^ influx remains elusive, through this review we hope to inspire more thoughts and discussion about how Ca^2+^ temporal profile differentially influences mitochondrial ROS production and cell death.

### Crucial Regulators of Ca^2+^ Homeostasis in Mitochondria

**FIGURE 1:**
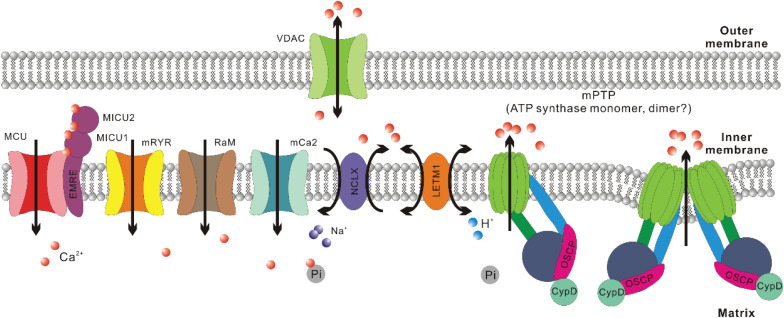
Crucial regulators of Ca^2+^ homeostasis in mitochondria. For mitochondrial Ca^2+^ uptake, the major routes include mitochondrial Ca^2+^ uniporter (MCU), mitochondrial ryanodine receptor (mRYR), rapid mode of mitochondrial Ca^2+^ uptake (RaM), and mitochondrial calcium channel type 2 (mCa2). The most crucial regulatory subunits of MCU include mitochondrial calcium uptake 1, 2 (MICU1, MICU2) and essential MCU regulator (EMRE). For mitochondrial Ca^2+^ extrusion, the major routes include Na^+^/Ca^2+^/Li^+^ exchanger (NCLX), mitochondrial H^+^/Ca^2+^ exchanger (LETM1), and mitochondrial permeability transition pore (mPTP). Voltage-dependent anion channel (VDAC) is suggested to be the outer mitochondrial membrane (OMM) component of mPTP. Recently, ATP synthase has been confirmed to be the inner mitochondrial membrane (IMM) component of mPTP. There are still debates about whether mPTP is formed by ATP synthase dimer or monomer. Cyclophilin (Cyp) D is a crucial regulator of mPTP opening and interacts with ATP synthase through the oligomycin sensitivity-conferring protein (OSCP) subunit. As to Ca^2+^ retention in mitochondrial matrix, inorganic phosphate (Pi) helps sequester free Ca^2+^ in solid precipitates, which could serve as an MCU independent source of mitochondrial Ca^2+^ upon matrix acidification.

This review does not intend to comprehensively cover all players involved in mitochondrial Ca^2+^ handling. However, a brief introduction is needed for meaningful discussions about the connections between [Ca^2+^]_mito_, ROS, apoptosis, and muscle atrophy (Figure 1).

Mitochondria are constantly involved in modulating spatiotemporal profiles of cytosolic Ca^2+^ ([Ca^2+^]_cyto_) in different types of cells under physiological and pathological conditions (Gunter et al., 2004; Szabadkai and Duchen, 2008; Demaurex and Guido, 2017), including cardiac and skeletal muscle (Zhou et al., 1998;Maack et al., 2006; Rizzuto and Pozzan, 2006; Sedova et al., 2006; Csordás and Hajnóczky, 2009; Yi et al., 2011). This is due to their abilities to uptake and extrude Ca^2+^, as well as retain Ca^2+^ in their matrix (Szabadkai and Duchen, 2008). Indeed, mitochondrial Ca^2+^ uptake has been first observed *in vivo* during skeletal muscle contraction induced by motor nerve stimulation (Rudolf et al., 2004) and later quantified in isolated individual muscle fibers during E-C coupling (Yi et al., 2011; Karam et al., 2017). The Ca^2+^ uptake routes in mitochondria include mitochondrial Ca^2+^ uniporter (MCU), mitochondrial ryanodine receptor (mRYR), as well as two other channels with unknown molecular nature: rapid mode of mitochondrial Ca^2+^ uptake (RaM) and mitochondrial calcium channel type 2 (mCa2) (Buntinas et al., 2001; Kirichok et al., 2004; Altschafl et al., 2007; Michels et al., 2009; Hoppe, 2010). In skeletal muscle, the presence of uptake routes other than MCU still waits to be confirmed. Overexpression of MCU in adult mouse flexor digitorum brevis (FDB) muscle led to notable enhancement in caffeine-induced mitochondrial Ca^2+^ influx as well as a moderate elevation of the resting [Ca^2+^]_m__i__t__o_ level (Mammucari et al., 2015). In contrast, transfection of FDB muscle with short hairpin (sh) RNA against MCU resulted in marked reduction of both resting [Ca^2+^]_m__i__t__o_ level and caffeine-induced mitochondrial Ca^2+^ influx (Mammucari et al., 2015). Furthermore, extensor digitorum longus (EDL) muscle infected by adeno-associated virus (AAV) carrying shRNA against MCU exhibited decreased pyruvate dehydrogenase (PDH) activity (Mammucari et al., 2015), which is known to be dependent on [Ca^2+^]_m__i__t__o_ level (Wan et al., 1989; Denton, 2009). Consistently, in skeletal muscles from MCU^–/–^ mice, resting [Ca^2+^]_m__i__t__o_ level seems reduced when compared to wild-type controls, while the phosphorylation of PDH, which is negatively correlated with the activity of Ca^2+^ sensitive phosphatase PDP1, significantly increased (Pan et al., 2013).

The activity of MCU is regulated by MICU1-MICU2 heterodimer, which senses [Ca^2+^]_cyto_ level (Mallilankaraman et al., 2012; Csordás et al., 2013; Kamer and Mootha, 2014; Patron et al., 2014; Wang et al., 2014). MICU1 is suggested to function as the cooperative activator of MCU, while MICU2 is believed to keep MCU closed at low [Ca^2+^]_cyto_ level (Patron et al., 2014). Skeletal muscle expresses a unique alternative splice isoform of MICU1, which is more sensitive to Ca^2+^ than the isoform expressed in other tissues and hence allows activation of MCU at a lower [Ca^2+^]_cyto_ level (Reane et al., 2016). This is likely an evolutionary adaption to the astounding amount of ATP consumed during muscle contraction since the activities of multiple enzymes involved in ATP synthesis are stimulated by Ca^2+^ (Wan et al., 1989; Das and Harris, 1990; Murphy et al., 1990; Denton, 2009; Glancy et al., 2013). Indeed, [Ca^2+^]_m__i__t__o_ transients in skeletal muscle are larger than those measured in other tissues based on mitoplast patch clamp results (Fieni et al., 2012). Other MCU regulatory subunits include EMRE and MCUb. EMRE helps tether MICU1 and MICU2 to the transmembrane region of MCU, while MCUb is a paralog of MCU that is suggested to negatively regulate MCU complex in a direct manner (Raffaello et al., 2013; Tsai et al., 2016).

The Ca^2+^ extrusion routes include Na^+^/Ca^2+^/Li^+^ exchanger (NCLX), mPTP and arguably mitochondrial H^+^/Ca^2+^ exchanger (LETM1) (Jiang et al., 2009; Hoppe, 2010; Palty et al., 2010; Nowikovsky et al., 2012). NCLX is more intensively expressed in skeletal muscle compared to many other tissues such as cardiac muscle (Palty et al., 2004, 2010). This is accompanied by an ultrafast Ca^2+^ efflux rate of skeletal muscle mitochondria, which is 2-3 orders faster than cardiac muscle (Rudolf et al., 2004; Palty et al., 2010). mPTP is a nonselective, large conductance megachannel mediating solute (<1.5 kDa) exchange between mitochondrial matrix and the outside milieu (Haworth and Hunter, 1979; Hunter and Haworth, 1979a, b; Kinnally et al., 1992; Szabó and Zoratti, 1992). The opening of mPTP is sensitive to Ca^2+^, cyclophilin (Cyp) D, oxidizing agents, thiol reagents, depletion of ADP, while its inhibitors include Mg^2+^, ADP, NADH, antioxidants, and cyclosporin (Cs) A (through interacting with CypD) (Haworth and Hunter, 1979; Hunter and Haworth, 1979a, b; Kinnally et al., 1992; Szabó and Zoratti, 1992). mPTP opening is widely known for its central role in cell death induction under multiple pathological conditions (Bonora et al., 2020). Osmotic influx of water into mitochondrial matrix through these pores results in swollen matrix, dissipated IMM potential and ceased ATP production. ATP dependent ion exchangers/pumps fail to maintain cellular ion homeostasis and finally lead to necrosis (Bonora et al., 2015). Additionally, mPTP opening has also been implied to facilitate the release of intermembrane space factors activating apoptotic pathway (Hirsch et al., 1997; De Giorgi et al., 2002; Borutaite et al., 2003). While the irreversible, high conductance mPTP opening upon [Ca^2+^]_mito_ overload is detrimental (Ichas et al., 1997; Hoppe, 2010; Karch and Molkentin, 2018), the transient and low conductance mPTP opening is considered a Ca^2+^ extrusion route that may carry out physiological functions (Ichas and Mazat, 1998; Elrod et al., 2010; Elrod and Molkentin, 2013).

The molecular composition of mPTP has remained elusive for over 60 years (Urbani et al., 2019). Previously mPTP was suggested to form from adenine nucleotide translocator (ANT) or phosphate carrier (PiC) on IMM, as well as voltage-dependent anion channel (VDAC) on OMM (Halestrap and Davidson, 1990; Szabó et al., 1993; Baines, 2009; Halestrap, 2009). However, further researches indicate that they are not the pore forming unit of mPTP, but could be regulatory components (Kokoszka et al., 2004; Krauskopf et al., 2006; Baines et al., 2007). In recent decades, CypD was reported to physically interact with oligomycin sensitivity-conferring protein (OSCP) within ATP synthase (complex V of OXPHOS) (Giorgio et al., 2009) and ATP synthase increases the permeability of IMM to different solutes upon [Ca^2+^]_mito_ overload (Giorgio et al., 2013, 2017; Alavian et al., 2014). However, the argument about whether it is the monomer or dimer of ATP synthase that carries out the megachannel function has not been settled yet (Mnatsakanyan et al., 2019).

The Ca^2+^ retention capacity of mitochondrial matrix is believed to heavily rely on the inorganic phosphate (Pi) entered mainly through mitochondrial phosphate carrier (PiC) (Szabadkai and Duchen, 2008; Seifert et al., 2015). Pi can buffer Ca^2+^ through the formation of osmotically neutral precipitates such as hydroxyapatite and whitlockite (Carafoli, 2010; Chinopoulos and Adam-Vizi, 2010), which theoretically should enable additional Ca^2+^ uptake through MCU, suppress Ca^2+^ efflux through NCLX and desensitize mPTP (Zoccarato and Nicholls, 1982; Szabadkai and Duchen, 2008; Seifert et al., 2015). However, these assumptions were challenged as opposite results were observed in PiC knockdown cells and PiC knockout mice (Varanyuwatana and Halestrap, 2012; Kwong et al., 2014). Meanwhile Pi is a long known sensitizer of mPTP (Zoratti and Szabò, 1995). Thus the precise role of the PiC in [Ca^2+^]_mito_ regulation still waits to be addressed. In addition, Ca–Pi precipitates can dissolve upon acidification and IMM potential disruption, enabling MCU-independent elevation of [Ca^2+^]_mito_ (Greenawalt et al., 1964; Wolf et al., 2017; Hernansanz-Agustín et al., 2020). For example, in cells experiencing acute hypoxia, complex I of OXPHOS in mitochondria undergoes conformational changes that lead to proton accumulation inside the matrix, dissolution of Ca–Pi precipitates, elevation of [Ca^2+^]_mito_ and activation of NCLX (Hernansanz-Agustín et al., 2020).

### Associations Between Steady-State Elevation of Mitochondrial Ca^2+^ Level and ROS Production

**FIGURE 2:**
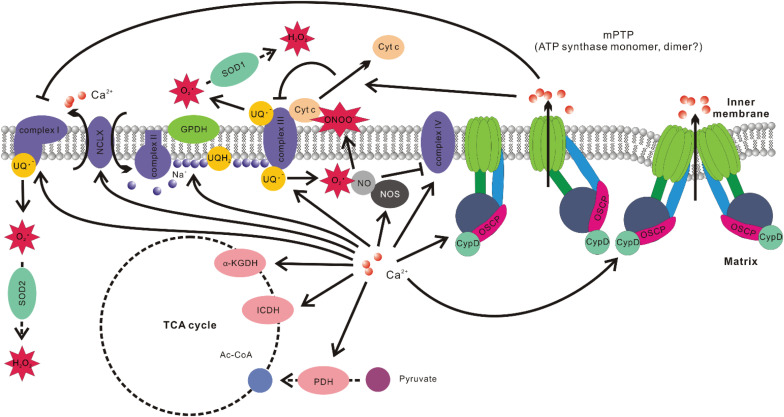
Associations between mitochondrial Ca^2+^ level and ROS production. Ca^2+^ promotes the activities of multiple Ca^2+^ sensitive dehydrogenase such as glycerol phosphate dehydrogenase (GPDH), pyruvate dehydrogenase (PDH), isocitrate dehydrogenase (ICDH), and α-ketoglutarate dehydrogenase (α-KGDH). It also elevates the efficiency of complexes I, III, IV, and V of OXPHOS. Ca^2+^-induced activation of NCLX results in Na^+^ influx into the matrix and Na^+^ interacts with phospholipid in the inner leaflet of IMM, decreases its fluidity, and slows down ubiquinol (UQH_2_) diffusion. This results in uncoupling of Q cycle and increased ROS production at *Q*_o_ site in complex III. Ca^2+^ stimulation of nitric oxide synthase (NOS) increases nitric oxide (NO) production, which inhibits complex IV by competing with O_2_. NO also readily react with superoxide to form peroxynitrite (ONOO^–^), which promotes cytochrome c (Cyt c) release. Cyt c release would disrupt the activity of complex III, increase the level of ubisemiquinone (UQ^–^), which provide one electron to O_2_ to form superoxide. Ca^2+^ is also the major stimulant of mPTP opening. The subsequent change of ion strength in mitochondrial intermembrane space disrupts electrostatic interactions between Cyt c and membrane lipid cardiolipin, leading to Cyt c release and hindrance of complex III activity. mPTP opening has also been implicated in inducing conformation changes of complex I, resulting in elevated ROS production.

Elevation of [Ca^2+^]_mito_ level could result in enhanced ROS production through multiple mechanisms (Figure 2):

1. Elevation of [Ca^2+^]_mito_ stimulates Ca^2+^ sensitive dehydrogenases, including glycerol phosphate dehydrogenase (GPDH), PDH, isocitrate dehydrogenase (ICDH), and α-ketoglutarate dehydrogenase (α-KGDH) (Wan et al., 1989; Denton, 2009). There is also evidence that Ca^2+^ increases activities of complexes I, III, IV, and V of OXPHOS (Das and Harris, 1990; Glancy et al., 2013). The faster O_2_ consumption rate results in increased ROS production under certain circumstances, although the opposite situation also exists (Barja, 2007; Neretti et al., 2009; Silva et al., 2009).

2. Elevation of [Ca^2+^]_mito_ stimulates mitochondrial nitric oxide synthase (NOS) to produce more nitric oxide (NO). NO can compete with O_2_ for binding sites on cytochrome c oxidase (complex IV of OXPHOS), which hinders the electron flow and decreases mitochondrial O_2_ consumption (Giulivi, 2003; Ghafourifar and Cadenas, 2005). The hindrance of the electron flow and the increase of local O_2_ may boost ROS production (Brookes et al., 2004). On the other hand, NO reacts readily with superoxide and generates peroxynitrite (ONOO^–^), which is a more potent ROS that causes Cyto c release, lipid peroxidation and oxidative damage to other vulnerable targets (Ghafourifar and Cadenas, 2005).

3. Elevated [Ca^2+^]_mito_ promotes the opening of mPTP. mPTP opening can lead to changes of ionic strength and hence disrupt the electrostatic interaction between Cyto c and cardiolipin in mitochondrial intermembrane space. Cyto c is required for the activity of ubiquinol-cytochrome c oxidoreductase (complex III of OXPHOS) (Ott et al., 2002). The blockage of complex III activity enhances ROS production by increasing the accumulation of the one-electron donor ubisemiquinone (Turrens et al., 1985; Muller et al., 2002, 2003). mPTP opening also seems to induce conformation changes in NADH-ubiquinone oxidoreductase (complex I of OXPHOS), resulting in increased ROS production (Batandier et al., 2004).

4. [Ca^2+^]_mito_ overload may stimulate Cyto c release from cardiolipin through competing for cardiolipin binding sites (Grijalba et al., 1999), which affects complex III activity and hence promotes ROS generation.

5. During acute hypoxia, elevation of [Ca^2+^]_mito_ due to matrix acidification activates NCLX, promoting the import of Na^+^. Matrix Na^+^ interacts with phospholipids in the IMM (such as phosphatidylcholine), reducing membrane fluidity, slowing down the diffusion of ubiquinol from glyceraldehyde 3-phosphate dehydrogenase (GAPDH) or complex II to complex III of OXPHOS, resulting in elevated ROS production of complex III at *Q*_o_ site (Hernansanz-Agustín et al., 2020).

### Transient Mitochondrial Ca^2+^ Influx Diminishes Denervation-Induced ROS Production and mPTP Opening in Skeletal Muscle

The cytosolic Ca^2+^ transients are spatiotemporally well-controlled Ca^2+^ release events from the sarcoplasmic reticulum (SR) responding to the motor nerve activation in a skeletal muscle fiber during excitation contraction (EC)-coupling. Skeletal muscle inactivity including neuromuscular diseases, spinal cord injury, and muscle unloading, etc., could partially or completely disrupt EC-coupling and eliminate cytosolic Ca^2+^ transients. A pathological hallmark of prolonged muscle inactivity is the enhanced ROS production in muscle fibers (Muller et al., 2007; Powers et al., 2012; Xiao et al., 2018). It is very well established that mitochondria are a major source of ROS production in prolonged muscle inactivity (Powers et al., 2012). Prolonged muscle unloading leads to an elevated resting [Ca^2+^]_cyto_ (Tischler et al., 1990; Ingalls et al., 1999), which in turn could overload mitochondria, increase the propensity of mPTP opening and stimulate ROS production (Csukly et al., 2006). However, the initial cause of mitochondrial ROS production in inactivated skeletal muscle remains elusive (Hyatt et al., 2019). One question is whether the cessation of physiological Ca^2+^ transients is an initiating factor for promoting mitochondria ROS production.

Transgenic mouse model expressing a mitochondria-targeted biosensor (mt-cpYFP) allowed a real-time measurement of a ROS-related mitochondrial signal, termed “mitoflash” (Wang et al., 2008; Ding et al., 2015). Although mitoflash activities could be composed of multifaceted signals including matrix alkalization, superoxide, and arguably some other oxidants (Wang et al., 2008, 2016; Schwarzländer et al., 2012; Wei-LaPierre et al., 2013), the mechanism underlying mitoflash events is believed to be linked to transitory opening of mPTP due to the high spatiotemporal correlations between mitoflash events and IMM depolarization (Wang et al., 2008, 2016). Besides transitory opening of mPTP, there are also other potential causes of IMM depolarization during mitoflash events, such as the activation of uncoupling proteins UCP2 and UCP3. However, inhibition of UCP2 by chemical blocker or RNA interference slightly increased, rather than decreased the mitoflash incidence (Wang et al., 2017). Skeletal muscle fibers derived from UCP3 knockout mice exhibited no changes in mitoflash frequency, amplitude or duration compared to wild type controls, while the average area of mitoflash events mildly reduced (McBride et al., 2019). Thus, these functional perturbation experiments do not support the hypothesis of UCP2/3 mediated uncoupling activities as the major contributor of mitoflash signals.

Using this transgenic mouse model, Karam et al detected a fourfold increase of mitoflash activity in skeletal muscle following a short period (24 h) of sciatic nerve transection (Karam et al., 2017). This denervation-induced mitoflash activity could be attenuated by the application of CsA, an established inhibitor of mPTP opening (Giorgio et al., 2018), further suggesting that mitoflash events reflect real-time opening of mPTP in denervated muscle fibers. Consistently, the increased mitoflash activity is associated with an elevated mitochondrial superoxide level reported with MitoSOX Red (Mukhopadhyay et al., 2007) in muscle fibers 24 h after the denervation. Similar results were also reported for muscle fibers derived from the ALS mouse model (SOD1^*G93A*^) at a stage before disease symptom onset (Xiao et al., 2018), when motor neuron axon terminal withdrawal (denervation) start to occur in individual muscle fibers (Frey et al., 2000; Fischer et al., 2004).

Due to lack of neuronal impulses, the action potential and physiological [Ca^2+^]_cyto_ transients are abolished in denervated skeletal muscle. Thus, a hypothesis was proposed that dynamic Ca^2+^ transients were required to keep mPTP at its closed state and maintain mitochondrial ROS production at the physiological level in skeletal muscle (Karam et al., 2017). Remarkably, when muscle fibers from denervated (24 h) mouse model were exposed to a brief electrical stimulation (40 Hz, 0.5 ms pulses at 8–12 V for a total duration of 350 ms) to restore physiological cytosolic and mitochondrial Ca^2+^ transients, the area and amplitude of mitoflash events dramatically reduced within a minute to a level comparable to the unstimulated sham muscle fibers (Karam et al., 2017). Importantly, after treating the denervated muscle fibers with Ru360 to block MCU for mitochondrial Ca^2+^ uptake, electrical stimulation no longer had significant impact on mitoflash activities. Additionally, the levels of mitochondrial superoxide (indicated by MitoSOX Red) also exhibited the same trend of changes under these conditions (Karam et al., 2017). Thus, mitochondrial Ca^2+^ influxes triggered by physiological cytosolic Ca^2+^ transients, even brief ones, seems to be capable of inhibiting transitory mPTP opening and ROS generation in mitochondria.

The above results are in line with previous discoveries that electrical stimulations help prevent apoptosis, retard muscle atrophy and improve muscle strength in denervated animal models (Mokrusch et al., 1990; Arakawa et al., 2010; Nakagawa et al., 2017) and patients with motor impairment caused by spinal cord injury or stroke (Dudley et al., 1999; Crameri et al., 2000, 2002; Doucet et al., 2012; Nascimento et al., 2014). However, the studies of the mechanisms underlying these phenomena usually focus on relatively long-term molecular changes, such as gene expression regulation, which usually takes tens of minutes to hours to occur (Voytik et al., 1993; Kostrominova et al., 2005; Arakawa et al., 2010; Peviani et al., 2010; Russo et al., 2010). The events occurred within a minute after electrical stimulation were rarely investigated.

One potential mechanism underlying the role of physiological Ca^2+^ transients in inhibition of mitochondrial ROS production could be that mitochondrial Ca^2+^ influxes induced by cytosolic Ca^2+^ transients suddenly boost the electron flow rate along the respiratory chain, decreasing the reduced state of ROS generators, such as ubisemiquinone generated by complex III of OXPHOS. Meanwhile, the sudden boost of respiratory chain activity also increases O_2_ consumption rate, decreasing the amount of local O_2_ available for forming superoxide. These two factors may both contribute to a quick attenuation of superoxide formation. Similar situation also occurs during sudden transition of state 4 respiration to state 3 respiration in isolated mitochondria (Barja, 2007).

**FIGURE 3:**
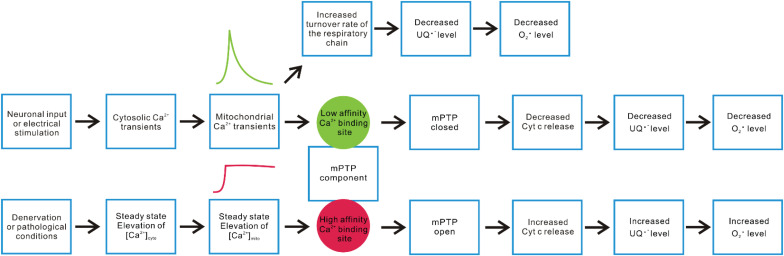
Hypothetical mechanisms underlying differential mitochondrial Ca^2+^ dynamics and ROS production. Neuronal or electrical stimulation-induced cytosolic Ca^2+^ transient can result in rapid Ca^2+^ influx into mitochondria, serving as a stimulant of multiple enzyme complex in OXPHOS that accelerate electron flux along the respiratory chain, leading to decreased accumulation of ubisemiquinone (UQ) and lower O_2_ partial pressure. Both factors contribute to alleviated superoxide production. On the other hand, we hypothesize that a key mPTP component may have two sets of Ca^2+^ sensory structures with different Ca^2+^ affinities, resulting in distinct responses to rapid versus steady-state elevation of [Ca^2+^]_mito_. Steady-state elevation of [Ca^2+^]_mito_ resulting from denervation or other pathological conditions may predominantly trigger the response mediated by a relatively higher affinity structure (such as the Ca^2+^ binding site within the F1 domain nucleotide binding pockets of ATP synthase), which promotes mPTP opening, enhances Cyt c release, disrupts complex III activity, and increases superoxide production. The rapid mitochondrial Ca^2+^ transients induced by motor neuron input or electrical stimulation may predominantly activate the response mediated by a relatively lower affinity Ca^2+^ responding structure, shutting down mPTP and decrease ROS production.

Additionally, as illustrated in Figure 3, we propose a second hypothetical mechanism that key mPTP components have two sets of Ca^2+^ responding structures with different affinities that trigger opposite changes in the propensity of mPTP opening based on the temporal profile of mitochondrial Ca^2+^ influxes in skeletal muscle fibers. Under this scenario, a steady-state increase of [Ca^2+^]_mito_ (a likely outcome of steady-state [Ca^2+^]_cyto_ elevation after denervation or neuronal degenerative disease) promotes mPTP opening through a relatively higher affinity Ca^2+^ responding structure. The second Ca^2+^ responding structure with relatively lower Ca^2+^ binding affinity may respond to the spike of mitochondrial Ca^2+^ influx following the cytosolic Ca^2+^ transient during EC-coupling activated by neuronal input or electrical field stimulation and quickly shut down the opening of mPTP in skeletal muscle. More than a coincidence, previous studies identified two binding sites for divalent cations (including Ca^2+^) on the F1 catalytic domain of ATP synthase. One located in the nucleotide binding pocket within the αβ cleft, the other located in the superficial loop of the β subunit and contains the acidic sequence DELSEED (Hubbard and McHugh, 1996; Giorgio et al., 2017, 2018). The first one is implicated as the “trigger site” of mPTP opening. More specifically, the occupancy of this site by Ca^2+^ (instead of Mg^2+^) is proposed to elevate the rigidity of F1 domain and transmit mechanical energy to OSCP, the peripheral stalk and finally to the IMM, leading to the formation of mPTP by ATP synthase dimers (Giorgio et al., 2017, 2018). Interestingly, the physiological role of the other Ca^2+^ binding site remains unknown. Due to the low affinity nature of this Ca^2+^ binding site, it may serve as the potential link between mitochondrial Ca^2+^ transients and quick mPTP shut down. It is also possible that the second Ca^2+^ responding structure locates on molecules other than ATP synthase, which acts as an accessory safe guard against mPTP opening upon Ca^2+^ binding during rapid mitochondrial Ca^2+^ influx. Further structural and functional studies are needed to validate those hypotheses. Nevertheless, the results reported in Karam et al. suggest that the physiological cytosolic and mitochondrial Ca^2+^ transients induced during EC-coupling are vital to keep mPTP in a closed status in skeletal muscle fibers.

### Exercise-Induced Signaling Involved in Mitochondrial ROS Regulation

**FIGURE 4:**
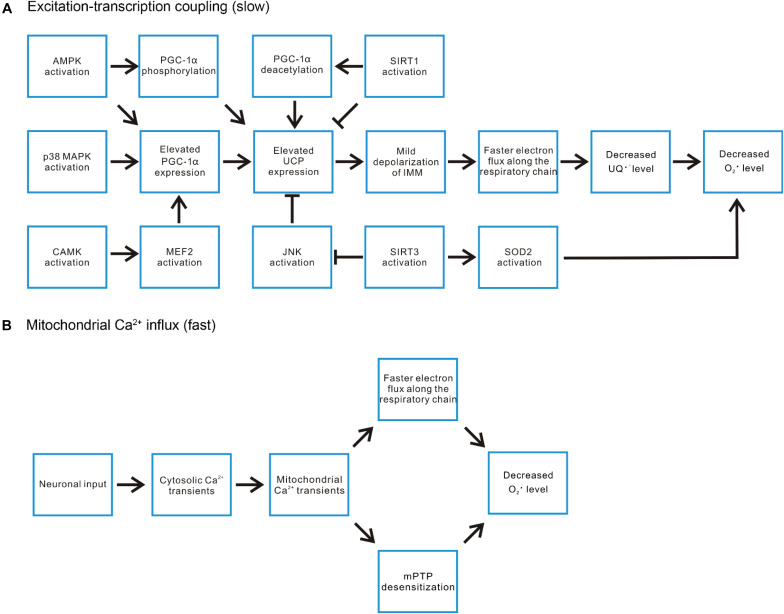
Exercise-induced signaling pathways regulate mitochondrial ROS level. **(A)** In the aspect of the excitation-transcription events, the ATP deficit during exercise induces AMPK activation, which can both directly phosphorylate and increase the expression of PGC-1α, the pivotal player in countering ROS-related damages. P38 MAPK and certain CAMKs have also been demonstrated to be activated by exercise and induce PGC-1α expression. SIRT1 activation promotes deacetylation of PGC-1α, enhancing its transcriptional activities. On the other hand, SIRT1 represses the expression of uncoupling protein (UCP2, UCP3). UCP induced mild depolarization of IMM accelerates electron flux along the respiratory chain, decreases the level of ubisemiquinone (UQ) and hence reduces superoxide production. Thus, the impact of SIRT1 activation on ROS level is mixed. In contrast, another SIRT family member SIRT3 promotes UCP expression by inhibiting JNK activation. It also promotes superoxide removal through deacetylating SOD2. **(B)** The rapid mitochondrial Ca^2+^ flux induced by motor neuron input may decrease ROS production through increasing the turnover rate of the respiratory chain or suppressing mPTP opening.

During exercise, muscle contraction dramatically increases ATP turnover rate, which could be more than 100-fold that of the basal rate (Hochachka and McClelland, 1997). To cope with such large demand of ATP, Ca^2+^ influx into mitochondrial matrix activates multiple enzymes related to TCA cycle and oxidative phosphorylation, elevating the synthesis of ATP in skeletal muscle (Wan et al., 1989; Das and Harris, 1990; Denton, 2009). The high O_2_ consumption rate and elevated [Ca^2+^]_mito_ level do not necessarily increase ROS production (Barja, 2007; Silva et al., 2009), which could be at least partially attributable to the exercise-induced signaling pathways, such as AMP-activated protein kinase (AMPK), protein kinase A (PKA), Ca^2+^/calmodulin-dependent protein kinase (CaMK), mitogen-activated protein kinase (MAPK), protein kinase C (PKC), focal adhesion kinase (FAK), and mammalian target of rapamycin (mTOR), cyclin-dependent kinase (CDK), integrin-linked kinase (ILK), and sirtuin (SIRT) family of protein deacetylases (Sakamoto and Goodyear, 2002; Egan and Zierath, 2013; Hoffman et al., 2015). Although some of these pathways are preferentially induced by acute exercise while the others mediate physiological adaptation to long-term endurance exercise, both of them involve transcriptional regulation and hence belong to excitation-transcription coupling framework (Egan and Zierath, 2013). It is beyond the scope of this review to systematically go through these pathways. But we would like to highlight some of them directly or indirectly involved in ROS regulation (Figure 4A).

The three best-characterized MAPK subfamilies are c-Jun N-terminal kinase (JNK), extracellular signal-regulated kinase (ERK), and p38 MAPK (Chen et al., 2001). All three subfamilies have been shown to be activated by oxidative stress (Torres and Forman, 2003; Li et al., 2005). Among them, p38 MAPK signaling has been reported to participate in contraction-induced PGC-1α gene expression in skeletal muscle (Akimoto et al., 2004). PGC-1α is a “master regulator” of mitochondrial biogenesis and promotes the expression of OXPHOS enzymes and uncoupling proteins (UCP2 and UCP3) (Wu et al., 1999; Zhou et al., 2000). Uncoupling proteins function to mildly depolarize IMM by increasing proton backflow into the matrix in a fatty acid (FA)-dependent manner (Brand and Esteves, 2005). This process could be described by an FA futile cycling model, in which UCP exports FA^–^ anions into intermembrane space. The anion diffuses away and gets protonated. Then, the protonated FA flip-flops across the membrane to deliver protons electro-neutrally back to the matrix (Jabůrek et al., 1999). UCP2 and UCP3 are not involved in thermogenesis adapting to cold temperature as UCP1 (Brand and Esteves, 2005). Instead, they are believed to serve as a cellular defense mechanism against superoxide formation, which works through accelerating the rates of proton pumping and electron flux along the respiratory chain, decreasing the level of one-electron donors (to O_2_) generated by complex I and III of OXPHOS (Brand, 2000; Zhou et al., 2000; Echtay et al., 2002; Brand and Esteves, 2005; Ježek et al., 2018). The expression of UCP3 is particularly high in skeletal muscle (Boss et al., 1997; Matsuda et al., 1997; Vidal-Puig et al., 1997; Liu et al., 1998) and is further induced by exercise or fasting (Gong et al., 1997; Tsuboyama-Kasaoka et al., 1998; Cortright et al., 1999). In contrast, denervation of skeletal muscle decreases the expression of UCP3 (Tsuboyama-Kasaoka et al., 1998).

AMPK can also be activated by ROS indirectly through ATP deficit, more specifically the increase of AMP/ATP and creatine/phosphocreatine ratios (Kahn et al., 2005; Irrcher et al., 2009). Acute exercise, due to the increased turnover of ATP, promotes AMPK activation in an intensity dependent manner (Wojtaszewski et al., 2000; Egan et al., 2010). AMPK activation has been report to mediate direct phosphorylation of PGC-1α and transcriptional upregulation of PGC-1α expression (Jäger et al., 2007; Irrcher et al., 2008). Meanwhile acute exercise induced UCP3 expression has also been demonstrated to involve AMPK (Zhou et al., 2000).

CaMKs are implicated in muscle fiber type switch adapting to long-term endurance exercise (Wu et al., 2000). Additionally, they have also been suggested to regulate mitochondrial biogenesis independent of fiber type transformation (Wu et al., 2002). Transgenic mice expressing constitutively active CaMKIV in skeletal muscle exhibited improved resistance to fatigue during repetitive contraction, augmented mitochondrial biogenesis, increased expression of OXPHOS genes (such as subunits of complex I) and PGC-1α (Wu et al., 2002). PGC-1α is a target gene of myocyte-specific enhancer factor 2 (MEF2), one of the transcription factors activated by CaMKIV (Passier et al., 2000). Furthermore, CaMKII has also been suggested to induce PGC-1α expression due to the activation of MEF2 (Liu et al., 2005; Egan and Zierath, 2013).

SIRTs are a family of protein deacetylases sensitive to elevated [NAD^+^] or NAD^+^/NADH ratio (Egan and Zierath, 2013). Their activities are closely linked with cellular metabolic status (Schwer and Verdin, 2008). SIRT1 mediated deacetylation is suggested to activate PGC-1α transcriptional activity on other genes (Gerhart-Hines et al., 2007; Gurd, 2011). However, SIRT1 also serves a potent repressor of UCP2 and UCP3 gene expression (Amat et al., 2007, 2009; Schwer and Verdin, 2008). Thus, SIRT1 exerts both positive and negative impact on ROS production. SIRT3 is a major deacetylase for mitochondrial proteins (Jing et al., 2011). In SIRT3 knockout mice, O_2_ consumption rate decreases while oxidative stress increases, accompanied by enhanced activation of JNK pathway (Jing et al., 2011). Consistently, knocking down SIRT3 in cultured myoblast also led to defective mitochondrial respiration capacity, increased ROS production and JNK pathway activation (Jing et al., 2011). Furthermore, SIRT3 has been shown to deacetylate SOD2, leading to increase in the SOD2 enzymatic activity to convert superoxide into hydrogen peroxide (Tao et al., 2014). Thus, the SIRT3 activity overall helps mitigate ROS accumulation (Tao et al., 2010, 2014).

It is also worth noticing that PGC-1α has an isoform termed PGC-1α4 resulted from alternative promoter usage and splicing (Ruas et al., 2012). This isoform is preferentially induced in mouse and human muscle during resistance exercise training (Ruas et al., 2012). Different from PGC-1α, it does not regulate OXPHOS genes but specifically induces IGF1 and represses myostatin, resulting in muscle hypertrophy (Ruas et al., 2012). Studies have indicated that the induction of PGC-1α4 requires MCU mediated Ca^2+^ uptake (Mammucari et al., 2015).

Although most studies concerning the beneficiary effect of exercise on ROS control focus on the excitation-transcription coupling framework, there is evidence directly linking acute exercise with improved mitochondrial functions such as decreased susceptibility to mPTP opening and increased O_2_ respiration rate in cardiac and skeletal muscle (Ascensão et al., 2011; Yoo et al., 2019a, b). These reports are consistent with the mechanisms we proposed underlying the instant decrease of mitochondrial ROS production in denervated skeletal muscle upon electrical stimulation. These two mechanisms may also explain why mitochondria were not the major contributor of ROS during electrical stimulation induced muscle contractions (Sakellariou et al., 2013). Thus, we propose that beside the excitation-transcription coupling framework, the rapid mitochondrial Ca^2+^ transients generated during exercise serve as a parallel mechanism contributing to prevent excessive ROS production, either through accelerating the turnover rate of the respiratory chain or suppressing mPTP opening (Figure 4B). The rapid mitochondrial Ca^2+^ influxes could also be one reason why there is no significant mitochondrial contribution to cytosolic ROS level in contractile skeletal muscle fibers.

## Summary and Future Perspectives

The elevations of [Ca^2+^]_mito_ induced by muscle contraction or muscle inactivity share many Ca^2+^ regulators in common, yet the outcomes are dramatically different in skeletal muscle ROS production. Additionally, electrical stimulation not only rapidly inhibits ROS production in an MCU dependent manner in skeletal muscle, but also prevents apoptosis, retards muscle atrophy in a longer term. These phenomena imply that [Ca^2+^]_mito_ temporal profile, likely in combination with steady-state [Ca^2+^]_mito_ level, serves as a toggle switch flipping between the beneficiary versus destructive outcomes. The players downstream of this toggle switch, aside from those relatively slower excitation-transcription coupling events, also include processes that modulate ROS production within the time frame of seconds to minutes. In this review, we proposed two potential mechanisms underlying this rapid process, such as direct regulation of the turnover rate of the respiratory chain and mPTP desensitization. Further experiments are needed to evaluate the validity of these hypotheses.

## Author Contributions

AL and JZ designed the scheme of this review. All authors contributed to editing the article and approved the submitted version.

## Conflict of Interest

The authors declare that the research was conducted in the absence of any commercial or financial relationships that could be construed as a potential conflict of interest.

## Abbreviations

AAV: adeno-associated virus
AIF: apoptosis-inducing factor
ALS: amyotrophic lateral sclerosis
AMPK: AMP-activated protein kinase
ANT: adenine nucleotide translocator
CaMK: Ca^2+^/calmodulin-dependent protein kinase
CDK: cyclin-dependent kinase
CsA: Cyclosporin A
CypD: cyclophilin D
Diablo: direct IAP (inhibitor-of apoptosis) binding protein with low pI
EC-coupling: excitation contraction-coupling
EDL: extensor digitorum longus
EMRE: essential MCU regulator
EndoG: endonuclease G
ERK: extracellular signal-regulated kinase
FAK: focal adhesion kinase
FDB: flexor digitorum brevis
GPDH: glycerol phosphate dehydrogenase
HtraA2: high temperature requirement protein A2
ICDH: isocitrate dehydrogenase
ILK: integrin-linked kinase
IMM: inner mitochondrial membrane
JNK: c-JunN-terminal kinase
MAPK: mitogen-activated protein kinase
mCa2: mitochondrial calcium channel type 2
MCU: mitochondrial Ca^2+^ uniporter
MICU: mitochondrial Ca^2+^ uptake
mPTP: mitochondrial permeability transition pore
mTOR: mammalian target of rapamycin
NCLX: Na^+^/Ca^2+^/Li^+^ exchanger
OMM: outer mitochondrial membrane
OSCP: oligomycin sensitivity-conferring protein
OXPHOS: oxidative phosphorylation
PKA: protein kinase A
PKC: protein kinase C
PDH: pyruvate dehydrogenase
RaM: rapid mode of mitochondrial Ca^2+^ uptake
PiC: phosphate carrier
RaM: rapid mode of mitochondrial Ca^2+^ uptake
ROS: reactive oxygen species
RYR: ryanodine receptor
SIRT: sirtuin
Smac: second mitochondria-derived activator of caspase
SR: sarcoplasmic reticulum.
